# Moderate prevalence of HIV-1 transmitted drug resistance mutations in southern Brazil

**DOI:** 10.1186/s12981-019-0219-1

**Published:** 2019-02-05

**Authors:** Larissa Danielle Bahls, Pedro Henrique Canezin, Edna Maria Vissoci Reiche, José Carlos Couto Fernandez, José Ricardo Colleti Dias, Vera Alice Fernandes Meneguetti, Luis Toshio Ueda, Dennis Armando Bertolini

**Affiliations:** 1 Department of Clinical Analysis and Biomedicine, State University of Maringá, Av Colombo 5790, Maringá, Paraná, CEP 87020-900 Brazil; 2 Department of Pathology, Clinical and Toxicological Analysis, State University of Londrina, Londrina, Brazil; 3 Laboratory of AIDS and Molecular Immunology, Oswaldo Cruz Institute, Rio De Janeiro, Brazil; 4 Service Specializing in STD/AIDS (SAE) of Maringá, Department of Health of Maringá, Maringá, Brazil; 5 Service Specializing in STD/AIDS (SAE) of Londrina, Department of Health of Londrina, Londrina, Brazil

**Keywords:** HAART, Genotype, Epidemiology, Mutation, Drug resistance

## Abstract

**Background:**

Despite the advances in therapy, the occurrence of drug-resistant human immunodeficiency virus type 1 (HIV-1) is a major obstacle to successful treatment. This study aimed to characterize the genetic diversity and to determine the prevalence of transmitted drug resistance mutations (TDRM) between individuals recently or chronically diagnosed with HIV-1 from Paraná, Brazil.

**Methods:**

A total of 260 HIV-1 positive antiretroviral therapy-naïve patients were recruited to participate on the study, of which 39 were recently diagnosed. HIV-1 genotyping was performed using sequencing reaction followed by phylogenetic analyses to determine the HIV-1 subtype. TDRM were defined using the Calibrated Population Resistance Tool program.

**Results:**

The HIV-1 subtypes frequency found in the studied population were 54.0% of subtype B, 26.7% subtype C, 6.7% subtype F1 and 12.7% recombinant forms. The overall prevalence of TDRM was 6.7%, including 13.3% for recently diagnosed subjects and 5.9% for the chronic group.

**Conclusions:**

The prevalence of resistance mutations found in this study is considered moderate, thus to perform genotyping tests before the initiation of antiretroviral therapy may be important to define the first line therapy and contribute for the improvement of regional prevention strategies for epidemic control.

**Electronic supplementary material:**

The online version of this article (10.1186/s12981-019-0219-1) contains supplementary material, which is available to authorized users.

## Background

Brazil has reported the largest number of acquired immune deficiency syndrome (AIDS) in Latin America [1]. The estimated number of people living with human immunodeficiency virus (HIV) in Brazil is 926,742, with sexual exposure being the main transmission path (96.9%), and predominance of the homo/bisexual category (48.7%). In the state of Paraná, in 2017, the detection rate was 17.1 per 100 thousand inhabitants, and, in the last 10 years, the total of accumulated cases was 15,699 [2].

Nevertheless, the rate of AIDS detection and mortality has been decreasing steadily in Brazil in recent years, mainly due to the antiretroviral therapy (ART), which is distributed by public health programs since the 1990s [2]. However, one unavoidable consequence of the widespread use of ART is the selection of mutations associated with drug resistance, which can be transmitted to uninfected individuals. Transmitted drug resistance mutations (TDRM) is associated with impaired outcomes from ART, including first line of highly active antiretroviral therapy (HAART) with two nucleoside reverse transcriptase inhibitors (NRTI) plus one nonnucleoside reverse transcriptase inhibitors (NNTRI) or one protease inhibitor (PI) [3] and other therapeutic interventions, such as pre-exposure prophylaxis (PrEP), post-exposure prophylaxis (PEP) and prevention of vertical transmission [4]. Detection of resistance mutations before the initiation of ART is considered cost-effective in areas where TDRM prevalence is 1% or higher, because it avoids spending public funds on drugs which will certainly result in virological failure [5].

In developed countries, guidelines recommend baseline genotypic testing in all patients newly diagnosed with HIV infection to ensure the effectiveness of first line ART [6, 7]. Indeed, Hofstra et al. demonstrated that in a setting without adequate surveillance, the prevalence of TDRM involving NNRTI resistant HIV can increase to worrisome levels, compromising the efficacy of standard first-line NNRTI-based regimen [8]. Unfortunately, although moderate levels of TDRM have been reported in Brazil [9–14], ranging from 3.8% in Maranhão [10] to 13.9% in São Paulo [12], the genotyping test is not available for most treatment-naïve patients [15].

Surveillance of TDRM among treatment-naïve recently diagnosed patient and of HIV-1 diversity is important, since it may contribute to the improvement of regional prevention strategies for epidemic control. Also, about 2 years after initial infection by HIV-1, the reversion of resistance mutations may occur; in other words, strains with TDRM can become undetectable in blood sample due to the growing of wild virus that has higher fitness than the mutated virus [16]. However, most studies developed in Brazil did not consider whether the patient was recently or chronically infected/diagnosed.

To the best of our knowledge, this was the first study that investigated HIV-1 diversity and evaluated TDRM between recently or chronically diagnosed ART-naïve patients with HIV-1 from Paraná State, Brazil.

## Materials and methods

### Study population

This is a cross-sectional study with consecutive sampling. A total of 281 individuals older than 18 years, seropositive for HIV-1 and treatment-naïve, who were attended at the Specialized Service STD/AIDS (SAE) of the municipalities of Maringá and Londrina, Paraná State, Brazil, from February 2010 to January 2013 were included in the study. The exclusion criteria were: co-infection with hepatitis B and/or C and history of ARV use. Based on this, the study population was formed by 260 subjects, which were divided into two groups, Recent and Chronic. The Recent group was composed by individuals whose first positive serology was dated less than 1 year from the date of collection for this study. To minimize the possibility of late diagnosis, patients with a CD4^+^ T cell count smaller than 400 cells/mm^3^ were excluded from the Recent group.

### Sample processing

Peripheral blood samples were collected with EDTA anticoagulant from all patients to determine viral and immunological parameters including counts of CD4^+^ T cells, viral load quantification and genotyping of HIV-1. Plasma was separated and kept at − 80 °C until use.

### CD4^+^ T cells counts

CD4^+^ T cells counts were performed using fresh total blood by flow cytometry (BD Trucount™ Tubes) using FACS Calibur device (Becton–Dickinson, NJ, USA). The results were expressed as cells/mm^3^.

### Viral load determination

Viral load was determined using plasma samples by branched DNA methodology (VERSANT^®^ HIV-1 RNA 3.0 Assay) with a System 340 bDNA Analyzer (Bayer Health Care, NY, USA). The results were expressed on a logarithmic scale in base 10.

### Genotyping of HIV-1

For HIV-1 genotyping, plasma viral RNA was extracted using the QIAmp^®^ Viral RNA Mini Kit (Qiagen, Courtaboeuf, France) extraction kit, according to manufacturer’s instructions, followed by retrotranscription reaction in order to obtain cDNA. The genes for protease (PR) and reverse transcriptase (RT) were amplified as described before [17] and the purified products of the polymerase chain reaction (PCR) were sequenced with ABI PRISM^®^ Kit BigDye™ Terminator version 3.1 (Applied Biosystems, Foster City, CA), according to manufacturer’s instructions, using the ABI 3500XL sequencer (Applied Biosystems, Foster City, CA).

Quality analysis and editing of the sequences obtained were performed in the SeqMan DNAStar Lasergene v7.0 program [18].

### Phylogenetic analysis and subtype determination

Subtypes were initially determined using Rega HIV Subtyping Tool v. 2.0 [19] and then confirmed based on the phylogenetic tree constructed in MEGA 5.0 software [20]. For the tree construction, sequences obtained from the Los Alamos HIV Reference Database were used [21]. Phylogenetic analysis was performed by a neighbor-joining algorithm using the Tamura-Neinucleotide substitution model, with 1000 replicates, the tree obtained is available as Additional file 1. The profiles of recombination were confirmed by bootscanning analysis with the Recombinant Identification Program v. 3.0—RIP 3.0 [22]. An additional file provide bootscanning results of the recombinants found in the study population (Additional file 2). Once HIV-1 has high genetic plasticity, sequences that presented ≥ 85% of similarity in phylogenetic analysis were excluded from the study to avoid genotyping error due to cross-contamination between samples.

### Transmitted drug resistance determination

Transmitted drug resistance was defined as the presence of at least one major mutation listed at Drug Surveillance Mutation Resistance (SDRM-2009). For this, the Calibrated Population Resistance Tool (CPR) v. 6.0 beta program was used [23]. The susceptibility of HIV to antiretrovirals was determined using the National Agency for AIDS Research (ANRS) algorithm v.22 [24].

### Statistical analysis

The prevalence of transmitted resistance was calculated based on the number of subjects in each group. Categorical variables were compared by two-tailed Chi square test or Fisher’s exact test, when applicable, with a confidence interval (CI) of 95%, using OpenEpi version 3.1 [25]. Quantitative variables were compared using the Mann–Whitney test using GraphPad Prism v. 5 (GraphPad Software Inc., San Diego, CA). All results of p value < 0.05 were considered statistically significant.

## Results

A total of 281 volunteers agreed to participate in the study; 91 of them were attended at the Maringá SAE and 190 at the Londrina SAE. Ten subjects were excluded due to co-infection and/or previous use of ARVs, and 11 were excluded due to the lack of data allowing classification of their infections as recent or chronic. Of the remaining 260 participants, 39 (15%) were classified as recently diagnosed and 221 (85%) as chronic carriers. There was no statistical difference between groups for gender, age and mode of transmission (Table 1).

**Table 1:** Epidemiological characteristics in treatment naïve individuals with HIV-1 from Paraná State, Brazil, 2010–2013

	Recent group (n = 39) *n (%)*	Chronic group (n = 221) *n (%)*	*p* value^*a*^
*Sex*			
Female	11 (28.21)	92 (41.63)	–
Male	28 (71.80)	129 (58.37)	0.1141
*Age*			
13–24	7 (17.95)	22 (9.96)	0.1133
25–49	28 (71.80)	163 (73.76)	0.4379
> 50	4 (10.26)	36 (16.29)	–
*Transmission*			
Sexual	36 (92.31)	208 (94.12)	0.7613
IDUs	1 (2.56)	8 (3.62)	–
Indeterminate	2 (5.13)	5 (2.26)	0.3747

*HIV-1* human immunodeficiency virus type 1, *IDUs* Injecting drug users

^a^Two-tailed Chi square test with confidence interval of 95%

The viral load levels found among individuals in the Chronic group (median = 4.61 log_10_; interquartile range = 3.79 to 5.05 log_10_) were significantly higher (p = 0.0004) than those found in individuals whose infection was considered recent (median = 3.88 log_10_; interquartile range = 3.54 to 4.37 log_10_). Regarding CD4^+^ T cell count, the average found in the Recent group (median = 583 cells/mm^3^; interquartile range = 502 to 807 cells/mm^3^) was significantly higher (p < 0.0001) than in the Chronic group (median = 317 cells/mm^3^; interquartile range = 95 to 503 cells/mm^3^).

**Fig. 1:**
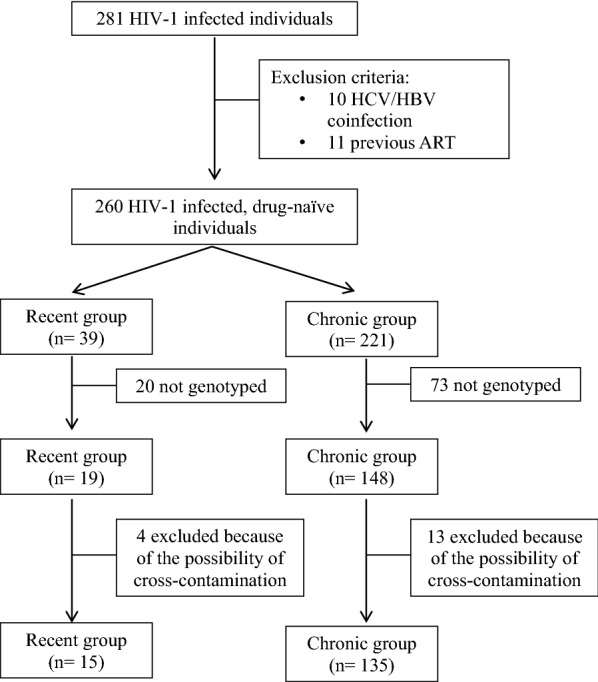
Flowchart of the study population

The samples of some subjects (93/260, 35.8%) were not amplified and sequenced. Additionally, to avoid genotyping error due to cross-contamination between samples, patients whose HIV-1 sequence presented ≥ 85% of similarity in phylogenetic analysis were excluded from statistical analyses. Thus, 150 samples were evaluated according to subtype and presence of ARV resistance mutations, 15 (10.0%) from the Recent group and 135 (90.0%) from the Chronic group (Fig. 1).

The phylogenetic analyses showed that most of the samples were subtype B (81/150, 54.0%), followed by subtype C (40/150, 26.7%), F (10/150, 6.7%) and recombinant forms (19/150, 12.7%) (Table 2), including BF (13/19, 68.4%), BC (1/19, 5.3%), BCF (3/19, 15.8%), BDF (1/19, 5.3%) and BCH (1/19, 5.3%).

**Table 2:** Frequency of HIV-1 subtypes and mutations associated with drug-resistance in drug-naïve individuals from Paraná State, Brazil, 2010-2013

	Recent group (n = 15)	Chronic group (n = 135)	All (n = 150)
*Subtype* ^a^			
B	10 (66.7; 41.7 to 84.8)	71 (52.6; 44.2 to 60.8)	81 (54.0; 46.0 to 61.8)
C	4 (26.7; 10.9 to 52.0)	36 (26.7; 19.9 to 34.7)	40 (26.7; 20.2 to 34.3)
F	0 (0.0; 0.0 to 20.4)	10 (7.4; 4.1 to 13.1)	10 (6.7; 3.7 to 11.8)
CRFs	1 (6.7; 1.2 to 29.8)	18 (13.3; 8.6 to 20.1)	19 (12.7; 8.3 to 18.9)
*Resistance mutations by class* ^b^			
PR region with PI mutation	0 (0.0; 0.0 to 20.4)	1 (0.7; 0.1 to 4.1)	1 (0.7; 0.1 to 3.7)
RT region with NRTI mutation	1 (6.7; 1.2 to 29.8)	3 (2.2; 0.8 to 6.3)	4 (2.7; 1.0 to 6.7)
RT region with NNRTI mutation	1 (6.7; 1.2 to 29.8)	4 (3.0; 1.2 to 7.4)	5 (3.3; 1.4 to 7.6)
Any resistance mutation	2 (13.3; 3.7 to 37.9)	8 (5.9; 3.0 to 11.3)	10 (6.7; 3.7 to 11.8)

Results are presented as N (%; 95% CI). HIV-1, human immunodeficiency virus type 1; PR, protease; RT, reverse transcriptase; PI, protease inhibitors; NRTI, nucleoside analogue reverse transcriptase inhibitor; NNRTI, non-nucleoside analogue reverse transcriptase inhibitors

^a^Comparisons performed using two-tailed Chi square test with confidence interval of 95%

^b^Comparisons performed using two-tailed Fisher’s exact test with confidence interval of 95%

All comparisons had *p* values > 0.05

The overall prevalence of TDRM was 6.7% (10/150), including 13.3% (2/15) in recently diagnosed patients and 5.9% (8/135) in the chronic group, with no significant difference between groups (p = 0.2764). Mutations associated with resistance to NRTIs were most frequently observed, followed by mutations associated with resistance to NNRTIs and only one sample presented TDRMs associated with resistance to PIs (Table 2). The mutations found in the study population are listed on Table 3 according to the HIV-1 subtype and time of diagnosis. One newly diagnosed patient showed two TDRMs for NRTIs and one subject of the chronic group had two TDRMs for PIs. No sequence was related to resistance to both NRTIs and NNRTIs or triple-class resistance.

**Table 3:** Drugs-resistance mutations according to HIV-1 subtypes and time of diagnosis in drug-naïve individuals from Paraná State, Brazil, 2010–2013

Sample identification	Diagnosis	HIV-1 subtype	NRTI mutations	NNRTI mutations	PI mutations	Resistance [23, 24]
L19	Chronic	B	–	K101E	–	EFV, NVP, ETR, RPV
L39	Chronic	C	–	K103 N	–	EFV, NVP
L97	Chronic	B	T215S	–	–	ZDV, d4T
L99	Recent	B	D67 N, T215S	–	–	ZDV, d4T, ABC
L150	Chronic	B	D67G	–	–	–
L156	Chronic	B	K70R	–	–	DDI, ZDV, d4T
L168	Chronic	C	–	G190E	–	EFV, NVP
M6	Chronic	C	–	K103 N	–	EFV, NVP
M18	Recent	B	–	K103 N	–	EFV, NVP
M19	Chronic	B	–	–	I54 V, V82A	IDV, NFV, LPV/r

*HIV-1* human immunodeficiency virus type 1, *PR* protease, *RT* reverse transcriptase, *PI* protease inhibitors, *NRTI* nucleoside analogue reverse transcriptase inhibitor, *NNRTI* non-nucleoside analogue reverse transcriptase inhibitors, *EFV* efavirenz, *NVP* nevirapine, *ETR* etravirine, *RPV* rilpivirine, *ZDV* zidovudine, *d4T* stavudine, *ABC* abacavir, *DDI* didanosine, *IDV* indinavir, *NFV* nelfinavir, *LPV/r* lopinavir/ritonavir

## Discussion

The transmission of resistant HIV-1 has important implications for the success of ART, since it restricts therapeutic options and increases the possibility of therapy failure. For this reason, this phenomenon has attracted the attention of researchers around the world. To our knowledge, this is the first study on the prevalence of transmitted resistance in the state of Paraná which considered the time of HIV-1 infection, discriminating patients recently diagnosed from those who have chronically established infections. The overall prevalence of transmitted resistance found in this study was 6.7%, with a prevalence of 13.3% among individuals with recent infection and 5.9% in chronically infected individuals. Regarding subtypes of HIV-1, subtype B was the most frequently found (54.0%), followed by subtype C (26.7%), and recombinant forms (12.7%). Subtype F had the lowest frequency in the study population (6.7%).

The increasing access to ART has caused a significant rise in rates of TDRMs [26]. The prevalence of TDRMs found in different studies varies according to the region and the population studied [27–31]. Based on the WHO HIVDR classification [32], the present study demonstrated a moderate level of resistance, which is in accordance to a recent survey conducted by Arruda et al. that revealed moderate rate of TDRM in the five Brazilian macroregions [33]. Sax et al. reported that in places where the prevalence of transmitted resistance is 1% or higher, testing for resistance before starting ART is considered cost-effective [5]. The Ministry of Health of Brazil recently published a document recommending the conduction of pretreatment genotyping only for HIV infected people with a current or previous partner taking ART, pregnant women, people co-infected with tuberculosis and children [15]. While in developed countries the prevalence of transmitted resistance is well surveilled, in Brazil the tests are not available for every HIV infected subject and studies on this aspect are few and conflicting.

This study found a TDRM prevalence of 6.7% among treatment-naïve individuals from the northern and northwestern regions of Paraná. This prevalence was similar to that found by Gaspareto et al. (4.2%) in northwestern Paraná in 2012 [17] and to that found by other Brazilian studies [10, 11, 14, 34–37]. Besides local social factors as the access to ART, the differences between the results reported in Brazilian studies for the prevalence of transmitted resistance among treatment-naïve individuals is due, mainly, to heterogeneity in study design, which occurs in the selection of the target population (patients with acute, recent or chronic infections), the methodology used to evaluate resistance mutations, and the definition of resistance mutations [16].

Among the TDRMs (5.9%) detected in this study, the most frequent were NNRTIs (2.9%), then for NRTIs (2.4%) and PIs (0.6%), which is similar to the ratio observed in other studies [35, 38]. However, these results differed from those of Sprinz et al. and Murillo et al., who found a higher frequency of TDRMs for NNRTIs [11] and from other studies that found a higher frequency of TDRMs for NRTIs, then for NNRTIs and for PIs [13, 34]. The K103N TDRM was the most frequently found in this study and has been described in other studies conducted in different regions of Brazil [13, 33–35, 38]. The presence of this mutation is associated with high levels of resistance to NNRTI Nevirapine and Efavirens, which were part of the recommended regimen for initial therapy in HIV-1 infections at the time of sample collection. Other mutations found for NNRTIs, K101E, also described by Pilotto et al. [13], and G190E are also associated with resistance to Nevirapine and Efavirens. All of the mutations that confer resistance to NRTI found in the present study, T215S, D67 N, D67G and K70R, are related to resistance to one of the first anti-HIV drugs approved in Brazil, Zidovudine, and have been observed in other Brazilian studies [13, 35, 38]. The I54 V and V82A mutations found in this study confer resistance to the PIs Indinavir, Nelfinavir and Lopinavir/Ritonavir. The current Brazilian therapeutic guideline recommends the association of two NRTI/NRTIt–lamivudine (3TC) and tenofovir (TDF)—with an integrase inhibitor (INI)—dolutegravir (DTG)—in the basis of the initial treatment scheme as an attempt to avoid the transmitted resistance problem [15].

Studies on the molecular epidemiology and genetic diversity of HIV-1 are essential to better understand the HIV-1 epidemic in Brazil. The frequency of HIV-1 subtypes varies according to the geographical region [39, 40]. The epidemiological profile of the different subtypes of HIV-1 in the country shows a clear predominance of subtype B, corresponding to about 75% of infected individuals [33, 36, 41–43]. Subtypes F and C are present in frequencies that differ from one region to another [36, 42, 43]. Subtypes A and D were also identified in isolated cases in Rio de Janeiro, although their epidemiological importance is not known yet [44, 45]. The frequencies for the different subtypes found in this study are in agreement with those found in other studies in southern Brazil [17, 46].

The present study has some limitations. The sampling of patients in care rather than consecutively of newly diagnosed individuals may have reduced the representativeness of the newly infected patients. Regarding the procedure used to classify the groups, no laboratory technique was used to determine if the infection was recent or chronic. However, viral and immunological parameters related to the course of infection, such as CD4^+^ T cells and HIV viral load, differed significantly between the groups, with a higher CD4^+^ T cell count in the Recent group and a higher viral load in the Chronic group, confirming that the criteria used were effective. Another limitation was the large number of unamplifiable samples, resulting in a high failure rate (36%). All the failed samples presented low or undetectable virus load. Additionally, the “in house” PCR methodology may have collaborated to the low sensitivity of the test.

## Conclusion

The present study demonstrated a moderate prevalence of TDRMs in the study population, which highlights the need to perform genotyping pretreatment tests to optimize the prescription of ART and prevent treatment failure due to a pre-existing mutation. Prospective studies are still needed to assess the true effectiveness of combination antiretroviral therapy for the treatment of infections with different subtypes of HIV-1 in Brazil. Knowledge on the resistance and cross-resistance of different subtypes of HIV-1 profiles will be critical to the choice of possible therapeutic options, particularly in Brazil, which currently provides antiretroviral therapy to nearly 90% of infected individuals. Additional investigations, including other gene targets, will be necessary to detect possible resistance mutations against new classes of antiretrovirals.

## Additional files

**Additional file 1.** Maximum likelihood phylogenetic tree of HIV* pol* (PR/RT) sequences obtained from ART-naïve patients from North and Northeast of Paraná, Brazil.

**Additional file 2.** Bootscanning analysis of recombinant HIV-1 sequences in protease and reverse transcriptase regions from ART-naïve patients from North and Northeast of Paraná, Brazil.

## Abbreviations

HIV-1: human immunodeficiency virus type 1
TDRM: transmitted drug resistance mutations
AIDS: acquired immune deficiency syndrome
ART: antiretroviral therapy
HAART: highly active antiretroviral therapy
NRTI: nucleoside reverse transcriptase inhibitors
NNTRI: nonnucleoside reverse transcriptase inhibitors
PI: protease inhibitor
PrEP: pre-exposure prophylaxis
PEP: post-exposure prophylaxis
PR: protease
RT: reverse transcriptase
PCR: polymerase chain reaction
