# Supplementary figures and images for: Moderate prevalence of HIV-1 transmitted drug resistance mutations in southern Brazil
Source: AIDS Res Ther. 2019 Feb 5;16:4. doi: 10.1186/s12981-019-0219-1 (PMC6364409; doi:10.1186/s12981-019-0219-1)

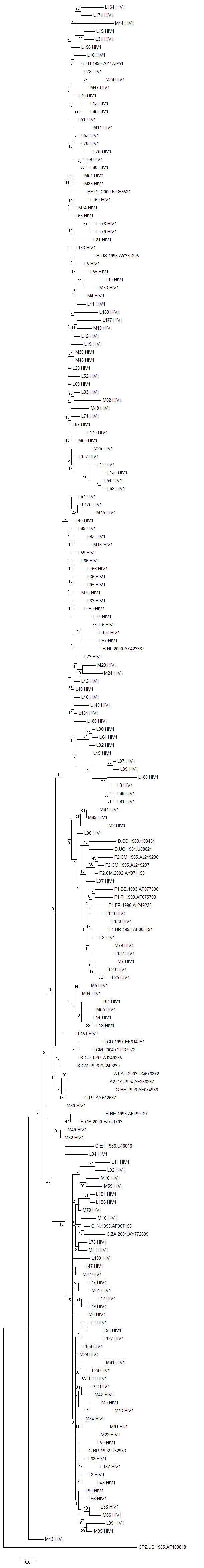

Supplement: Supplementary file 1 — Additional file 1. Maximum likelihood phylogenetic tree of HIV pol (PR/RT) sequences obtained from ART-naïve patients from North and Northeast of Paraná, Brazil. [file 12981_2019_219_MOESM1_ESM.tif]

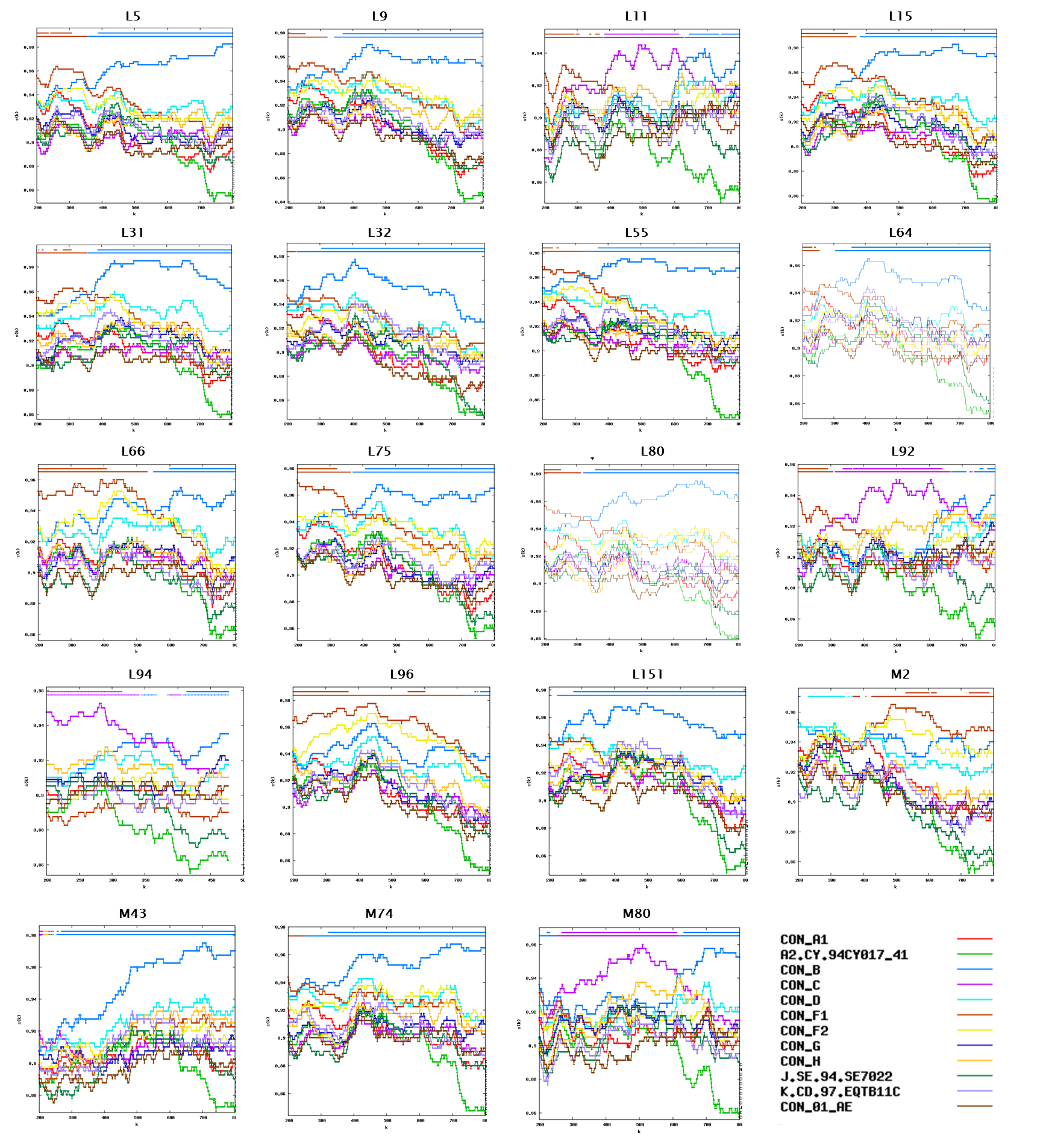

Supplement: Supplementary file 2 — Additional file 2. Bootscanning analysis of recombinant HIV-1 sequences in protease and reverse transcriptase regions from ART-naïve patients from North and Northeast of Paraná, Brazil. [file 12981_2019_219_MOESM2_ESM.tif]
